# Commencements

**Published:** 1894-08

**Authors:** 


### COMMENCEMENTS.

### University of Minnesota—Dental Department.

The annual commencement exercises of the Dental Department of the University of Minnesota were held, in connection with those of the other departments of the University, at Minneapolis, Minn., June 7th, 1894.

The degree of D. M. D. (Doctor of Dental Medicine) was conferred on the following graduates:

| NAME. | RESIDENCE. | NAME. | RESIDENCE. |
|---|---|---|---|
| John Paul Handy | Long Prairie. | Alfred Owre | Minneapolis. |
| Martin Franklin Lowe | Fairmont. | James Martin Walls | St. Paul. |
| Frank Herman Mero | Minneapolis. | Arthur Deming Whiting | Northfield. |

Total, 6.

### Chicago College of Dental Surgery.

The twelfth annual commencement exercises of the Chicago College of Dental Surgery (Dental Department of Lake Forest University) were held in the Columbia Theater, Chicago, Ills., on Monday, April 30th, 1894, at 2 P. M.

The number of matriculates for the session was three hundred and sixty.

The address to the graduates was delivered by John M. Coulter, Ph. D., LL. D., President of the University, and the valedictory by Geo. H. Denison.

The degree of D. D. S. was conferred on the following graduates by Truman W. Brophy, M. D., D. D. S., Dean of the Faculty:

| NAME. | RESIDENCE. |
|---|---|
| Frank H. Baker | Illinois |
| Carl O. Bauth | Illinois |
| Fred. H. Burkhardt | Illinois |
| John R. Brown | Illinois |
| Herbert B. Cole | Michigan |
| Edward H. Czolbe | Illinois |
| Arthur P. Davis | Wisconsin |
| Geo. H. Denison | Indiana |
| Arnold E. Dick | Hungary |
| Orlan T. Eddy | Illinois |
| Walter W. Eberhart | Minnesota |
| Geo. H. Edgerton | Illinois |
| Eno L. Fickensher | Illinois |
| William C. Gowan | Canada |
| Robt. C. Gebhardt | Wisconsin |
| Joseph E. Grattelo | Illinois |
| Geo. W. Harris | Minnesota |
| Frank R. Houston | Wisconsin |
| Alfred L. Haas | Iowa |
| David A. Hare | Canada |
| Ralph W. Harold | N. Dakota |
| Albert M. Harrison | Illinois |
| Fred. J. R. Hemple | Michigan |
| Edward H. Hickman | Illinois |
| Chas. W. James | Illinois |
| John W. Johnson | Canada |
| John K. Kane | Illinois |
| Carl R. Kuderling | N. Dakota |
| Geo. R. Lee | Illinois |
| B. J. La Grange, M. D. | California |
| Samuel E. Leece | Wisconsin |
| Elsie H. Leib | Illinois |
| Chas. C. Mann | Washington |
| Alex. O. McBean | Wisconsin |
| Arthur D. O'Neill, Jr. | Illinois |
| John E. Pollock | Illinois |
| Milton B. Pine | Indiana |
| Ceylon Rossman | Minnesota |
| Jasper M. Revel | Canada |
| Ruger C. Stevenson | Wisconsin |
| Frederick B. Skillman | Illinois |
| William F. Saunders | Wisconsin |
| Chester G. Stinson | California |
| Thomas Sheridan | Illinois |
| Floyd Sykes | Wisconsin |
| Frank Smith | Illinois |
| Alfred B. Sargent | Minnesota |
| Chas. L. Thomas | California |
| Lewis P. Voss | Illinois |
| George W. Winslow | Indiana |
| Chas. G. R. Wright | Illinois |
| John J. Wright | Wisconsin |
| Joseph S. Wright | Mississippi |
| Henry L. Whipple | Illinois |
| Simon F. Wilhelmi | Illinois |
| Thompson P. Wheat | Illinois |
| Chas. M. Wesner | Illinois |
| Charles H. Wylie | Wisconsin |

Total 58.

### Northwestern Uuiversity Dental School.

The annual commencement exercises of the Northwestern University Dental School were held at the Central Music Hall, Chicago, Ill., on Tuesday, April 24, 1894.

The number of matriculates for the session was ninety-five.

The degree of D.D.S. was conferred on the following graduates:

| NAME. | NAME. |
|---|---|
| Lucian H. Arnold | William Lansing Lewis |
| William Spencer Bagley | Charles Casper Lipe |
| Samuel Augustus Beel | Fred. William Marriett |
| Edwin Kenton Bennington | Paul George Maxon |
| Leander Read Brown | Abraham Louis Oberndorf |
| Louis Arthur Edwards | Caspar Paul Pfaff |
| Charles Herbert Eldred | Hosea Edwin Reid |
| Benjamin Franklin Ells | Aurin Ralph Shaw |
| Frederick Wilhelm Fritsche | Edwin Henry Smith |
| Robert Leslie Hopkins | Charles Mackay Smith |
| Philip Freeman Kittoe | Claude Bryant Warner |
| Louis Ladewick | Herbert Devere Wilson |

Total, 24.

### University of Buffalo—Dental Department.

The second annual commencement exercises of the Dental Department of the University of Buffalo were held, in connection with those of the Medical and Pharmaceutical Departments, in Music Hall, Buffalo, N. Y., Tuesday evening, May 1, 1894.

The number of matriculates for the session was eighty-six.

The examinations before the Board of Curators, comprising the State Dental Examining Board, were held during the day, and at the close the following candidates for graduation were recommended to the Chancellor of the University for the degree of Doctor of Dental Surgery, which was accordingly conferred upon them:

| NAME. | NAME. |
|---|---|
| Arthur G. Bullock | Joseph L. Povall |
| Joseph W. Beach | Frank L. Sibley |
| Seymour E. MacDougall | William H. Snider |

Total, 6.

### University of California—College of Dentistry.

The annual commencement exercises of the College of Dentistry of the University of California were held at Odd Fellows' Hall, San Francisco, Cal., Thursday evening, June 14, at eight o'clock.

The address on behalf of the Faculty was delivered by Joseph D. Hodgen, D.D.S., '87.

The degree of D.D.S. was conferred on the following graduates by Martin Kellogg, A.M., LL.D., President of the University:

| NAME. | NAME. |
|---|---|
| Edward Ellsworth Baird, | William O'Rourke, |
| William Henry Cavill, | Henry Powell, jr., |
| McCoy Chappell, | William Judson Taylor, |
| Conrad Deichmiller, | Leander Van Orden, jr., M.D., |
| John Alvin Gibbon, | Thomas Augustus Vogel, |
| Gilbert Fuller Graham, | David John Vogelman, |
| Robert Frederick Gray, | William Henry Wadsworth, |
| Walter Simpson Jewell, | Walter Irving Wilcox, |
| James William Likens, | Charles Calvin Wood. |

Total, 18.

### Harvard University—Dental Department.

The annual commencement exercises of the Harvard Dental School were held, in connection with those of the other schools of the University, in Sanders Hall, Cambridge, Mass., on Wednesday, June 27th, 1894.

The number of matriculates for the session was sixty-three.

The degree of D.D.S. was conferred upon the following graduates by the President of the University, Charles William Eliot, LL.D.:

| NAME. | NAME. |
|---|---|
| Eugene Everett Arnold, | Thomas Edward Quinn, |
| Joseph Bergin Belliveau, | Fred. Gibson Robbins, |
| Joseph Boylston, | Arthur Galusha Smith, |
| Jay Reuben Holton, | George Lund Taft, |
| Joseph Inderbitzen, | Louis Napoleon Veo, |
| Arthur Jackson, | William Joseph Walton. |
| Frederick William Percival, | |

Total, 13.

### NOTICES.

### The American Dental Society of Europe.

Notice has already been given in the July number of the DENTAL REGISTER, of the nineteenth meeting of the American Dental Society of Europe, which is to be held in the *Athenee*, Geneva, Switzerland, August 6th, 7th and 8th inclusive.

The following list of papers to be read and discussed has been prepared, viz.: "A Few More Words on Pyorrhœa," by Dr. A. V. Elliott, of Florence; "Theory of the Crystal Mat

